# Stem Cell-Based Cell Carrier for Targeted Oncolytic Virotherapy: Translational Opportunity and Open Questions

**DOI:** 10.3390/v7122921

**Published:** 2015-11-27

**Authors:** Janice Kim, Robert R. Hall, Maciej S. Lesniak, Atique U. Ahmed

**Affiliations:** The Department of Surgery and the Brain Tumor Center, The University of Chicago, Chicago, IL 60637, USA; janice.k1004@gmail.com (J.K.); rrhall3@uchicago.edu (R.R.H.); mlesniak@surgary.bsd.uchicago.edu (M.S.L.)

**Keywords:** cell carrier, oncolytic virus, stem cell

## Abstract

Oncolytic virotherapy for cancer is an innovative therapeutic option where the ability of a virus to promote cell lysis is harnessed and reprogrammed to selectively destroy cancer cells. Such treatment modalities exhibited antitumor activity in preclinical and clinical settings and appear to be well tolerated when tested in clinical trials. However, the clinical success of oncolytic virotherapy has been significantly hampered due to the inability to target systematic metastasis. This is partly due to the inability of the therapeutic virus to survive in the patient circulation, in order to target tumors at distant sites. An early study from various laboratories demonstrated that cells infected with oncolytic virus can protect the therapeutic payload form the host immune system as well as function as factories for virus production and enhance the therapeutic efficacy of oncolytic virus. While a variety of cell lineages possessed potential as cell carriers, copious investigation has established stem cells as a very attractive cell carrier system in oncolytic virotherapy. The ideal cell carrier desire to be susceptible to viral infection as well as support viral infection, maintain immunosuppressive properties to shield the loaded viruses from the host immune system, and most importantly possess an intrinsic tumor homing ability to deliver loaded viruses directly to the site of the metastasis—all qualities stem cells exhibit. In this review, we summarize the recent work in the development of stem cell-based carrier for oncolytic virotherapy, discuss the advantages and disadvantages of a variety of cell carriers, especially focusing on why stem cells have emerged as the leading candidate, and finally propose a future direction for stem cell-based targeted oncolytic virotherapy that involves its establishment as a viable treatment option for cancer patients in the clinical setting.

## 1. Introduction

**Figure 1:**
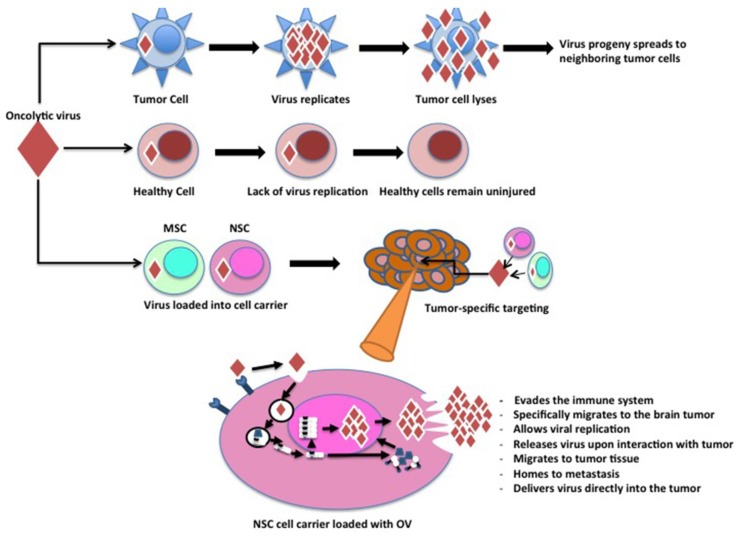
Comparison of modified virus behavior in healthy cells and tumor cells along with mesenchymal stem cells (MSCs) and neural stem cells (NSCs). Virus modification inhibits the ability of virus replication in healthy cells, eliminating the potential of widespread infection upon oncolytic virotherapy injections. However, injected viruses rapidly replicate in tumor cells, causing lysis of tumor cells. This allows the virus to destroy malignant cells and spread throughout the tumor population.

Oncolytic virotherapy is an uprising approach for treating human malignancy. Such treatments either utilize genetically engineered virus or virus with natural tropism towards transformed cells to induce targeted destruction of tumor burden by replication-mediated killing [1,2]. Evolution of these designer viruses to target cancer is founded on the attractive notions that oncolytic virotherapy not only has the potential to be efficacious even after delivery of small doses of therapeutic virus to the tumor burden via tumor selective replication mediated amplification of the therapeutic payload, but most importantly these viruses have the capacity to distinguish between normal and cancerous tissues (Figure 1). Administration of these smart viruses has proven to be safe both in pre-clinical and clinical trials and has demonstrated a moderate to high level of anti-tumor activity in experimental animal models [3]. Unfortunately, the therapeutic efficacy of many tested oncolytic viruses (OVs) in clinical trials have been limited due to various immunological, physiological and intratumoral barriers [4,5].

Most cancers that lack therapeutic options usually presented with disseminated metastasis, and thus require systemic therapy. In ideal conditions, treatments for patients with metastatic cancers would give targeted therapy such as oncolytic virotherapy systemically, allowing the injected viruses to locate and destroy tumor cells at distant sites without affecting healthy tissues. However, significant challenges remain in targeting systemic disease with “naked” (unprotected therapeutic virus particles in the circulation) therapeutic viruses administered into the patient’s circulation. Host immune system-mediated neutralization is evoked during the first week of therapy and ablates the delivery of repeated systemic administration of naked therapeutic virons [6]. Additionally, intratumoral barriers such as hypoxia, connective tissue compartments and rapid proliferation and outgrowth of non-targeted tumors also create issues that reduce the therapeutic efficacy of oncolytic virotherapy [7,8].

Since clinical applications of OV have issues in targeting systemic diseases, a “Trojan Horse” strategy was proposed to shield therapeutic payloads in the circulation while they home toward tumor beds[9,10]. This concept has evolved from the natural infection process, where it was observed that once the virus enters the host circulation the majority of the infected viruses usually are rapidly eliminated by the host immune system. However, a very small amount of virus can be sequestered by passerby cells and can be protected from immune-mediated neutralization. For example, human immunodeficiency virus (HIV) can bind to dendritic cells (DC) and utilize the natural migratory sites of DC to the lymph nodes in order to infect CD4 T cells [11]. Based on this natural precedent of viral infection, researchers demonstrate that a cell can also function as vehicle for therapeutic virus [9,10,12]. These observations have led to a decade-long investigation to develop a cell-based carrier for anti-cancer gene and virotherapy. In such dynamic biotherapy, a cell can function as a vehicle to deliver the therapeutic payload to distant tumor sites by protecting them from host immune surveillance as well as preventing the uptake by the off-target organ. Many different cell types have been extensively evaluated as vehicles for oncolytic virotherapy. In this review, we will discuss qualities of an ideal cell carrier while summarizing previously published studies regarding the stem cell-based cell carrier and its potential to be a better carrier system for oncolytic virotherapy.

## 2. Characteristics of an Ideal Cell Carrier Oncolytic Virotherapy

Therapeutic virus loading on a carried system is usually carried out *ex vivo* with one leading objective: to package as many OV onto or into the carrier system as possible. This objective is crucial, as the loading dose is typically directly proportional to the therapeutic dose available at the tumor sites. Moreover, loading of the therapeutic virus must occur rapidly, as any premature initiation of OV replication will not only reduce the viability of the cell carrier, but will also increase the likelihood of untimely presentation of the viral antigen at the surface of the cell carrier and thus the virus will be eliminated by the host immune system.

Secondly, a complete cell carrier must have some degree of ability to defend the therapeutic payload from the host’s immune system. Oncolytic virotherapy has the greatest potential to be successful in the clinical setting if such therapy can be administered systemically to target the metastatic tumor burden effectively. This approach holds a significant challenge, as unprotected “naked” viral particles in the circulation are highly vulnerable to immune recognition [13]. The immune system has evolved to protect us from foreign pathogens, but does not have the complexity to distinguish between therapeutic and pathogenic viruses. Therapeutic virus delivery into the circulation triggers a near immediate response from the host immune system, which leads to neutralization of the therapeutic payload within 30 minutes [14]. Furthermore, a majority of the population carries pre-existing antibodies against various oncolytic vectors such as adenovirus and measles virus [15,16]. These anti-viral antibodies mediate a rapid neutralization of therapeutic cargo present in the patient circulation, leading to significant reduction of the therapeutic dose at the tumor site [6]. One way to augment the therapeutic dose at the tumor site is to repeatedly administrate the therapeutic virus, but this approach can produce therapy-induced neutralizing antibodies that severely reduce the efficacy of systemic oncolytic virotherapy [17]. In the animal model, systemic administration of adenovirus vectors generated neutralizing antibodies within ten days of initial therapy, with these antibodies reaching plateau level in 2–3 weeks. To effectively translate oncolytic virotherapy in the clinical setting, OVs must avoid immune recognition and achieve prolonged survival in the circulation. Thus, an ideal candidate for the carrier system must offer a capability to function as “Trojan Horse” in order to protect the therapeutic payload from the host immune response.

Most importantly, an effective carrier system must possess some degree of intrinsic tumor homing ability. Once the OVs are delivered into patient circulation, cell carriers must be able to navigate through the hostile environment to locate tumors at distant sites and selectively deliver the therapeutic cargo. Recently, different cell systems have been evaluated as cell carriers--with a few of them exhibiting varying magnitudes of tumor homing capacity (Summarized in Table 1.) Mechanistically, the tumor homing ability of these carriers has been associated with the specific characteristics concerning the tumor cells, the tumor microenvironment with secreted soluble factors, and the anatomical location of the specific tumor type [5,6]. For clinical success, a successful cell carrier must have the capacity to home to the tumor. Therefore it is critical to carefully characterize the cell carrier types via their homing patterns as well as to predict their distinct migratory behaviors once they are in the circulatory system (Table 1).

**Table 1:** Examination of cell lineages used as carriers for delivery of oncolytic viruses to malignancies. Advantages and disadvantages of each type of carrier are indexed, with citations of preclinical studies investigating these carriers listed adjacently.

Type of Carrier	Advantages	Disadvantages	Reference
Transformed Cancer Cells			
Solid Tumors	Often stimulate antitumor immunity. Support rapid replication of the viruses they carry. Easy to inject.	Large size limits which tumor forms they can treat. Can cause new metastases. Administer low amounts of virus because of immediate immune responses upon injection.	[18,19,20,21]
Hematopoietic and lymphoid tumors	Kinesis via the circulatory system.	Rapid proliferation rate can lead to *de novo* tumors. Elicit immune response, reducing amount of virus delivered.	[15]
Xenogeneic/allogeneic	Injected cells are destroyed, preventing *de novo* metastases.	Immune response is profound, limits delivery because or side effects and rejection of injected cells.	[6]
Immune Cells			
T cells	Home to metastases. Activated at tumor cite, release virus specifically into tumor. Do not elicit immune response.	Strong preference to be loaded with reoviruses. Usually refractory to viral infection *in vivo.*	[22,23,24]
Activated T Cells	Increased ability to take up viruses. Efficacy of viral treatments increases.	Activation is lengthy and tedious. Do not support all viruses.	[25,26]
CIKs	Home to tumors. Release high amounts of viruses upon reaching the tumor. Can affect a variety of tumor types.	Requires expansion of primary leukocytes using cytokines *in vivo*.	[27]
Progenitor Cells			
Blood outgrowth endothelial cells	Very targeted delivery because of ability to become incorporated into tumor neovasculature Divide successfully and rapidly *in vivo.*	Cells are not immortal, new cells must be isolated from clinical samples. Currently unknown if they can support infection with replicating therapeutics.	[28]
Mesenchymal Stem Cells	Migrate to the tumor tissue. Allow viral replication. Release virus upon interaction with tumor. Evade the immune system.	High amount of non-specific migration in some cancers. Must be harvested from bone marrow.	[29,30]
Neural Stem Cells	Specifically migrate to brain tumors. Allow viral replication. Evade the immune system.	Require stereotactical extraction of cells from the subventricular zone.	[31,32,33]

Finally, once it arrives at the tumor site, an ideal cell carrier must function as a virus-producing factory by supporting therapeutic virus replication. Thus, each viral vector must unite with the optimal cell carrier system to function affably while targeting the metastatic tumor burden. For example, some OVs are engineered to express viral genes essential for replication controlled by tumor-specific promoters [34]. For a cell to effectively function as a carrier system for OVs, it is crucial that it maintains high tumor specific promoter activity and supports viral replication. A cell with these specific characteristics will be the ideal cell carrier for oncolytic virotherapy.

## 3. Stem cell as Cell Carrier

In recent years, stem cells have received extensive attention regarding their therapeutic potential for various diseases. Seminal studies have demonstrated that stem cells possess an inherent tropism toward invasive malignancies in experimental animal models [35,36,37,38]. Even though the molecular mechanisms and the purpose for such tropic behavior is still under investigation, the discovery of stem cells’ ability to home to tumors following systemic or distance administration instigated a novel platform to develop targeted therapies for invasive and metastatic malignancies [34,38,39]. In recent years, numerous studies have demonstrated the feasibility of stem cell carriers by pairing them with different biological agents, ranging form suicide genes to therapeutic nanoparticles, and demonstrating anti-tumor activity [34,40,41,42]. In this section, we will discuss different characteristics of stem cells in order to rationalize their use as carriers for oncolytic virotherapy.

**Figure 2:**
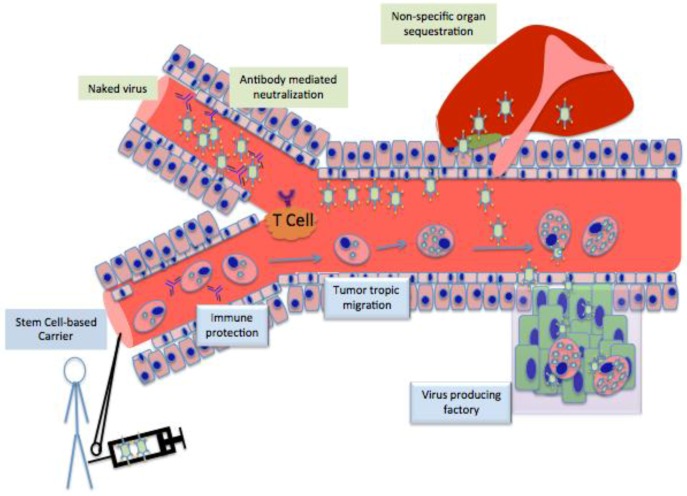
Antibodies react to the injection of naked viruses, prohibiting viruses from reaching the tumor site because of filtration by the liver. Contrastingly, injections of stem cells carrying modified viruses evade antibodies and T Cells because of the low amount of transporters associated with antigen processing (TAP) in stem cells. Stem cell carriers are not filtered to the liver and home directly to the tumor site. Upon reaching the tumor, stem cell carriers act as replication factories for viruses, prompting the release of numerous viruses directly into the tumor.

*GPS to locate the systemic and invasive tumor burden:* The intrinsic tumor homing ability of a variety of stem cells has been well documented in the literature; this specific characteristic makes stem cells to be most fitting of all the carrier systems for the use in oncolytic virotherapy (Figure 2) [34,35,36,37,38,39] To date, various growth factors, chemoattractant molecules and their cogent receptors have been investigated as drivers for stem cells’ ability to home to tissue injuries. These investigations have indicated SDF1/CXCR4, VEGF/VGFFR, HGF/c-Met, SCF/c-kit, and TGFb/TGFbRII signaling pathways are responsible for such migration [43,44,45,46,47]. Experimental conclusion asserts that the intrinsic tumor homing properties of stem cells are mediated by very similar mechanisms. The tumor microenvironment expresses various growth factors, angiogenic factors, cytokines and chemokines to support uncontrolled tumor growth. These pro-tumorigenic molecules have the potential to generate a gradient surrounding the tumor milieu that can function as a chemoattractant, causing stem cells to home to outlying tumor sites. For example, low oxygen tension, or hypoxia, is one of the common features of many cancers that regulates different pro-tumorogenic growth factor expressions. Early investigation indicated that tumor hypoxia is critical for promoting tumor-tropic migration of different cells, including stem and progenitor cells [48,49]. The hypoxia-inducible factor (HIF), a master transcription factor that regulates hypoxia, also promotes expression of stromal cell-derived factor-1 (SDF-1), vascular endothelial growth factor (VEGF) and urokinase plasminogen activator (uPA) in glioma cells, which is also critical in determining neural stem cells’ (NSCs’) tropism towards glioblastoma tumors, while antibody-mediated blocking of their cogent receptor signaling significantly abolished their tumor-tropic behavior [49,50]. Hypoxia-regulated IL-6 plays a critical role in survival and recruitment of mesenchymal stem cells (MSCs) to the hypoxia milieu within the tumor [51].

Viral infection can rapidly induce innate immune responses in the host cells by altering various intracellular signaling pathways, which can also influence the cell motility [52,53]. Thus, limiting the effects that *ex vivo* loading of individual therapeutic viruses into stem cell-based cell carriers has on their tumor homing ability will be critical in determining the ability of stem cells to function as cell carriers for oncolytic virotherapy. In our experience, loading of the glioma tropic oncolytic adenovirus CRAd-S-pk7 into the immortalized NSC lines HB1.F3.CD or ReNcells did not alter their tumor homing ability deleteriously [34,50]. Moreover, at an optimal loading dose of 10–50 infectious units (i.u.)/NSC the therapeutic viruses significantly enhanced their directional migration towards GBM both *in vitro* and *in vivo* in the orthotopic glioma xenograft model [50]. Our study indicated that loading with the OV enhances expression of chemoattractant receptors, such as CXCR4 and VRGFR2, which leads to enhanced homing capacity [50]. On the other hand, oncolytic adenovirus loading into mesenchymal stem cell (MSC)-based cell carriers resulted in downregulation of CXCR4 and c-MET expression and enhanced their non-specific migratory capacity [31]. Shinojima *et al.* reported that the tumor homing ability of the bone marrow-MSC is regulated by TGFβ/TGFβRII signaling [47]. In our experience, the CRAd-S-pk7 loading into MSCs resulted in significant upregulation of TGFβ expression but did not augment their tumor homing ability [31]. This may be due to autocrine production of the chemoattractant factor TGFβ post OV loading, which may saturate the legend-receptor signaling, resulting in decreased of the directional migration but enhancement of non-specific migration. For NSC-based cell carriers, the regulation of tumor homing ability is arbitrated by dynamic interplay between multiple signaling pathways such as SDF1/CXCR4 and VEGF/VGFFR [49,50]. Such multifaceted tumor homing mechanisms employed by stem cells may errand the use of one cell carrier over another, predominately in the one-dimensional targeting strategy such as antibody mediated tumor targeting. Eventually, a careful examination and optimization will be crucial for each OV to maximize its ability to influence the stem cell-based carrier to effectively target metastatic cancer in the clinical setting.

*Protect the therapeutic payload from immune surveillance:* Immune responses against the viral vector seem to be one of the critical roadblocks preventing clinically relevant therapeutic efficacy of systemically administered oncolytic virotherapy against metastasis (Figure 2). When a therapeutic virus is loaded into a cell carrier, an effective carrier system must act to delay the antigen processing and presentation of the viral proteins. Many published reports demonstrated that stem cells are highly immunosuppressive and inadequately immune stimulatory [32,54]. Overall, stem cells express lower levels of tapsin (TPN) along with fewer transporters associated with antigen processing 1 (TAP-1), both of which are crucial for antigen processing and presentation [8]. Bone marrow-derived MSCs express lower levels of MHC class I and have the ability to inhibit both T-cell proliferation and differentiation of monocytes into dendritic cells (DCs), induce the toleragenic phenotype of the effector T cells, and suppress expression of interferon-gamma along with tumor necrosis factor produced via CD4 T-helper cells [54,55]. Thus, MSCs serve as viral reservoirs with the potential to become causative factors of transplantation-related complications [56]. On the other hand, NSCs express a very high level of MHC class I/II in symmetry to co-stimulatory molecules such as CD80 and CD86 [32,57]. Such occurrence results in inefficient killing and clearance of NSCs by the alloreactive cytotoxic T cells and natural killer cells. These characteristics are very appealing attributes of stem cells for their use as cell carriers in oncolytic virotherapy, which may allow this carrier system to protect the therapeutic payload from host immune surveillance as it travels through the circulation to locate the metastatic/invasive tumors at distant sites.

We have investigated the ability of bone marrow-derived MSC (BM-MSC) in the immunocompetent cotton rat model to protect oncolytic adenovirus and demonstrated that virus loaded MSCs can persist longer in tumors as compared to naked viruses since the host’s antiviral immune response is significantly dampened [58]. The *ex vivo* loading of the oncolytic adenovirus into BM-MSCs as well as NSCs activated innate immune responses within 16 to 32 h post-loading and induced expression of IL6, INFγ, TNFα, RENTES and MCP1 [31]. However, surprisingly, the BM-MSCs and NSCs expressed the immunosuppression molecules TGFβ and IL10 respectively after being infected with the oncolytic adenovirus. Most importantly, intracranial delivery of therapeutic virus loaded into stem cell-based cell carriers not only significantly reduced the immune response measured by MHC Class II and inhibited the presence of reactive GFAP positive astrocytes as compared to delivery of the naked virus, but also allowed the therapeutic virus to persist much longer at the tumor sites, resulting in enhanced therapeutic efficacy [31,34,50]. These studies support the hypothesis that the immunosuppressive property of stem cell-based cell carriers may provide a novel platform to circumvent the host antiviral immune response in oncolytic virotherapy.

## 4. Different Stem Cells as Cell Carrier for Oncolytic Vitrotherpy

Stem cells from various sources have been investigated as cell carriers for oncolytic virotherapy, including mesenchymal stem cells (MSCs), neural stem cells (NSCs) and adipose-derived stem cells. Each type of stem cell has its advantages and disadvantages with respect to their ability to function as a cell carrier. The approaches for testing the efficiency of stem cell carriers can vary, yet the conclusion asserts that the stem cell-based cell carrier approach is a viable approach to deliver OVs for treatment of tumors. Here we focus on the two most widely used stem cells, MSCs and NSCs (Summarized in Table 2), which have been tested extensively to determine their effectiveness as cell carriers for oncolytic virotherapy.

MSCs are multipotent stem cells that can be derived from many different tissues, and have the capacity to give rise to a variety of cell types [67]. MSCs possess the ability to migrate to tumor tissue along with the capability to release and amplify loaded viruses by supporting replication of different OVs and protecting them from immune-mediated neutralization [31,42,62,64,68,69,70,71]. One of the most commonly used oncolytic viral vectors is human adenovirus serotype 5, which uses the Coxsackie adenovirus receptor (CAR) for entry into the target cells, there establishing infection [72]. The *ex vivo* OV loading into the cell carrier is directly related to the expression of the CAR receptor on the surface of the cell carrier system. Even though BM-MSCs express sufficient amounts of adenovirus entry receptor CAR, transductional modifications of oncolytic adenovirus with 5/3 fiber chimerism significantly enhance loading efficiency without altering migratory ability, indicating that such modification can be employed in oncolytic virotherapy even if the stem cell carrier expresses a low level of the entry receptor [50,59,73]. Engraftment efficiency of stem cells is a major challenge in the cell replacement therapy [67]. To circumvent this problem, Shah and collogues recently demonstrated that oncolytic adenoviruses and herpes simplex viruses loaded into MSCs can be encapsulated in biocompatible synthetic extracellular matrix (sECM), which significantly improved retention of the cell carrier at the resection cavity as well as enhanced the therapeutic efficacy in the orthotopic glioma xenograft model [14,74,75]. However, it is yet to be determined how such encapsulation might alter the intrinsic homing ability of MSCs. Moreover, there are conflicting reports regarding the tumor homing ability of MSCs. Hakkarainen *et al.* examined MSCs derived from bone marrow and adipose tissue for their ability to function as oncolytic adenovirus cell carriers and demonstrated that retargeted oncolytic adenoviruses can efficiently infect and replicate in MSCs derived from both types of tissue [73]. However, their study raised a question regarding the specificity of MSCs’ tumor homing ability, as they show that systemic administration of MSCs loaded with OV can preferentially accumulate in the lung. Despite such non-specific migration, intravenous delivery of MSCs loaded with oncolytic adenoviruses was able to prolong the survival of animals with orthotopic lung cancer as compared to a naked virus. Peng and colleagues demonstrated that systemic administration of adipose tissue-derived MSCs from patients with ovarian cancer could rapidly home to systemic tumor burdens [64]. Moreover, treatments using measles virus loaded MSCs can significantly increase the survival of animals with systemic ovarian cancer as compared to naked virus therapies. In another track of a potential OV candidate, Yong *et al.*, reported that MSCs loaded with adenovirus Delta24-RGD halted tumor growth when injected, increasing survival from 42 days to 75.5 days in an *in vivo* murine glioma xenograft model. The inference was that MSCs loaded with Delta24-RGD OV have the ability to specifically target human gliomas and deliver the therapeutic payload to the tumor sites effectively. In our experience, loading of oncolytic adenoviruses can alter the chemoattractant receptor expression and promote nonspecific migration of MSCs as compared to NSCs in the CNS. Taken together, these data indicate that different stem cells may be appropriate for targeting different types of cancer and thus require extensive preclinical evaluation for their homing abilities before they can be tested in the clinical setting.

**Table 2:** Summary of preclinical trails using stem cells for oncolytic virotherapy treatments. Studies investigating mesenchymal stem cells are listed first, followed by studies examining neural stem cells. The type of virus loaded and cancer treated are catalogued. Results and citation of each preclinical trial are also included.

Type of Stem Cell Carrier	Species of Origin	Type of Virus	Type of Cancer Treated	Result	Reference
Bone marrow-derived mesenchymal stromal cells	Human	Oncolytic Adenovirus	Pancreatic	Capsid modification leads to enhancement of therapeutic viral loading onto MSC-based cell carriers (Engineered 5/3 fiber chimerism adenoviruses enter MSCs at a 35-to 3310-fold rate compared to adenovirus 5 wild type capsid.)	[59]
Bone marrow-derived mesenchymal stem cells	Human	Osteocalcin promoter-directed Ad-hOC-E1 oncolytic adenovirus	Renal Cell Carcinoma	Injection of pharmaceutical inducible MSC carrying oncolytic adenovirus combined with vitamin D_3_ treatment induced effective viral delivery to RCC tumors and significant tumor regression. These were significantly greater than those of injection of carrier-free Ad-hOC-E1.	[60]
Bone marrow-derived mesenchymal stem cells	Human	Adenovirus carrying the *IFN-*β gene	Glioblastoma	MSCs home to tumors in murine models. MSCs loaded with therapeutic virus injected intra-arterially prolonged median survival of animals.	[61]
Bone marrow-derived mesenchymal progenitor cells	Human	Adenovirus Ad5/3	Ovarian	MSCs home to ovarian tumors, allow virus to replicate, and prolong survival *in vivo.* (Median survival time of 34 days for mice treated with PBS controls, 44 days for the uninfected MPC transplanted controls, but 69 days in the oncolytic virus-infected MPCs group.)	[62]
Bone marrow-derived mesenchymal stem cells	Human	Adenovirus Delta24-RGD	Glioblastoma	Carotid injections of MSCs loaded with therapeutic eradicated tumors, halted tumor growth, and prolonged survival. (Increase in median survival from 42 days to 75.5 days in murine *in vivo* models with autotrophic patient derived glioma.)	[63]
Mesenchymal stem cells derived from ovarian cancer patients (ovMSC)	Human	Measles	Ovarian	Migration of ovMSCs to tumors was comparable to that of MSCs derived from healthy donors. Delivery of virus *in vivo* using mice passively immune to measles yielded similar results upon treatment with MSCs and ovMSCs, both of which elicited longer survival than naked measles virus injection alone. (Median survival for PBS control is 36 days, 37 days for MV injection, but 82 days for MSC/MV injection.)	[64]
Bone marrow-derived mesenchymal stem cells	Human	Measles	Hepatocellular Carcinoma	Systemically delivered MSCs homed to HCC tumors implanted in the liver. MSCs effectively transferred MVs via heterofusion. The therapy inhibited tumor growth in passively immunized SCID mice, which did not occur upon naked MV injections.	[34]
Immortalized fetal brain-derived neural stem cells	Human	Adenoviral vector CRAd-S-pk7	Glioblastoma Multiforme	Viral loaded NSC therapy, when delivered prior to, rather than after conventional therapy prompts 30% longer survival in mice with autotrophic patient-derived glioma compared to application after therapy. (Adenoviral loaded NSC injections in conjunction with XRT-TMZ treatments increased median murine survival 46% compared to XRT-TMZ alone.)	[65]
Immortalized neural stem cell type HB1.F3-CD derived from fetal brain	Human	Adenoviral vector CRAd-S-pk7	Glioblastoma Multiforme	Virus delivered via NSC carrier was localized within the injected hemisphere. NSC carrier cells handed off the therapeutic virus to tumors within 5 days post-injection *in vivo* in mice with autotrophic patient-derived glioma.	[66]
Human fetal brain-derived neural Stem Cells	Human	Adenovirus	Glioblastoma Multiforme	NSCs are superior viral cell carriers to MSCs in targeting glioma. NSCs release virus at an amount a log higher than MSCs (*p* < 0.001). NSCs injected intracranially in an orthotropic glioma model increased the survival of tumor bearing animals more robustly than MSCs (median survival for NSCs 68.5 days against 44 days for MSCs, *p* < 0.002)	[31]

Another extensively evaluated stem cell type for oncolytic virotherapy is the NSC. NSCs are progenitor cells from the central nervous system (CNS) that can be isolated from the developing and adult CNS [76]. Our laboratory has been examining various types of NSC-based cell carrier systems to achieve selective delivery of a glioma tropic oncolytic adenovirus CARd-S-pk7 [31,34,50,77]. CRAd-S-pk7 is a conditionally replicating virus with the human adenovirus serotypes 5 (Ad5) backbone that expresses the wild type replicative essential E1A gene under the control of a tumor specific surviving promoter [78]. We have been evaluating the Food and Drug Administration approved immortalized neural stem cell line HB1.F3.CD as a cell carrier for oncolytic virotherapy (NCT01172964) [34,65,66]. HB1.F3.CD cells express very low levels of CAR and thus it is quite challenging to infect and load wild type Ad5 while using this cell line [34]. However, incorporation of poly-L-lysine (pk7) into the C-terminus of the wild-type fiber knob domain allowed us to achieve CAR-independent infection and efficient loading of CRAd-S-pk7 into HB1.F3.CD. Intracranial delivery of HB1.F3.CD loaded with CRAd-S-pk7 at the contralateral hemisphere of GBM implanted animal brains not only delivered the therapeutic virus to distant tumors, but also significantly prolonged animal survival as compared to a naked virus. The conventional anti-glioma therapies, radiation and chemotherapy, did not impede the NSC-based anti-glioma oncolytic virotherapy, but rather showed that these modalities can work together to improve the survival of animals with patient-derived glioma xenografts [65]. Taken together, these studies validate the notion that NSC-based cell carriers can be used for targeted delivery of OVs to invasive GBM and will serve as the foundation for an investigational new drug application for a human clinical trial in the near future.

In search for the optimal cell carrier for anti-glioma oncolytic virotherapy, we performed a preclinical compression of NSC and MSC using CRAd-S-pk7 virus in the orthotopic GBM xenograft model [31]. Our data indicated that at least in the CNS, NSC-based cell carriers demonstrate superior tumor homing abilities and prolong survival of animals with GBM xenograft more effectively than MSC-based carriers. Loading these cells with therapeutic viruses altered various chemoattractant receptors expression in both cell carriers and resulted in alteration of their migratory properties [31,34,50]. Innate immune responses that occurred post-loading of the therapeutic viruses resulted in the production of a number of immunostimulatory molecules as well as immunosuppressive molecules such as TGFβ and IL-10.

MSCs freshly isolated from patients are an attractive candidate for oncolytic virotherapy carrier system because the feasibility of using autologous cells may allow us to avoid allograft rejection. MSCs can be isolated from the bone marrow, adipose tissues and other tissues very efficiently, and the protocol to expand these cells *ex vivo* is fairly well established [67,79]. However, these freshly isolated MSCs can be very heterogeneous, making it difficult to predict their behavior, especially with respect to their tumor homing abilities and *ex vivo* loading capacities. Moreover, intra-patient variability for the amount and the quality of MSCs that can be isolated depend on patient age and current health status. In planning for a clinical trial, Mader *et al.* recently demonstrated that MSCs isolated from healthy donors and MSCs isolated from patients with new and recurrent ovarian cancer show very similar phenotypes and doubling times [64]. Within two weeks, these cells can be expended *ex vivo* to generate the amount of cells required for a clinical trial. However, the fact that about 20% of isolated MSCs show abnormal karyotypes is somewhat concerning, even though these MSCs were not tumorigenic in the immunocompromised animals. Additional studies characterizing freshly isolated heterogeneous MSCs with respect to their behavior post implantation would be beneficial in order to use these cells in the clinical setting.

On the other hand, NSCs have been evaluated as a carrier system to target malignancies in the CNS due to their origin and their inherent tumor tropism [35,37,77,80,81]. The majority of these studies utilized immortalized NSC lines and thus posses a logistical problem related to their immunogenicity as well as the risk of inducing secondary malignancies [80]. We have extensively examined the safeness of the immortalized NSC lines in the preclinical animal model, and we observed a very favorable safety profile with no sign of tumor formation in the immunodeficient animal brain after a year post NSC implantation [66]. Despite this, the concern regarding the possibility of secondary malignancy must not be overlooked, and additional safety measures such as further modification of these stem cell lines with suicide gene systems must be considered (Table 2).

## 5. What Next for Stem Cell-Based Cell Carrier?

With difficulties involving the delivery of the OV via the blood stream, the possible search of a new cellular vehicle for OV can raise hopes in improving the current status of oncolytic virotherapy. A lot of clinical data regarding the use of stem cells as cell replacement therapy has been recently generated [50]. These data must be carefully examined in order to successfully translate stem cell-based cell carrier to the clinical setting. In reviewing recent accomplishments of the usage of carrier cells for delivery of OVs, the future of stem cell carriers seems to be assessed on safety, feasibility and efficacy in human patients. It is crucial to have a better understanding of stem cell characteristics and to deepen knowledge of their behavior post implantation prior to testing the stem cell-based cell carrier for oncolytic virotherapy in the clinical setting. Next, we discuss some open questions and possible solutions of successful clinical translation of stem cell based cell carrier.

## 6. Limitations and How to Overcome Them

*Upgrading the GPS system:* The ability of stem cells to travel a great distance and home to systemic metastases and disseminated tumor burdens is fundamental to their utility as cell carriers for oncolytic virotherapy. Even though sufficient pre-clinical data in the rodent model demonstrate such characteristics, the distance that implanted stem cells have to travel in patient circulation may be considerably further than in any rodent system. Moreover, only 20%–30% of implanted NSCs were able to home to distant tumor sites [50]. The migratory NSC sub-population expresses elevated levels of VEGFR2 as compared to non-migratory NSCs and the VEGFA-VEGFR2 signaling pathway-mediated expression of MT1-MMP is critical for allowing the tumor homing abilities of NSCs [82]. By utilizing the precise molecular mechanism of their tumor homing capacity, stem cells can be engineered to further enhance their tumor homing abilities. Overexpression of the CXCR4 chemoattractant receptor on the NSC-based cell carrier significantly enhanced the tumor homing ability as well as the therapeutic efficacy of NSC-based oncolytic virotherapy (MS Lesniak and I Balyasnikova unpublished data). For stem cell-based cell carrier to be successful in the clinical setting, we must elucidate the molecular details of their intrinsic tumor homing abilities and utilize this knowledge to develop strategies to maximize the number of cell carriers that reach distant metastases.

*Allogeneic stem cell line*, *iPS and personalized cell carrier:* An *ex vivo* culture technology that has the capacity to isolate large quantities of autologous stem cells from a patient with the shortest amount of time would be ideal to avoid allogeneic implantation of stem cell-based cell carrier that might be subject to rapid rejection by the host immune system [83]. However, such optimized culture system is yet to develop. Thus, the possibility of using autologous MSCs as a cell carrier for OV is very attractive because it may allow us to avoid allograft rejection. However, it is difficult to predict the quality and quantity of MSCs isolated from a cancer patient who has received multiple rounds of chemotherapy. On the other hand, even though there has been extensive research supporting the safety of immortalized NSC cell lines in preclinical animal models, the risk associated with secondary malignancy cannot be overlooked. With that said, newly developed induced pluripotent stem cells (iPS) provide an opportunity to develop a personalized cell carrier for each patient, which will allow us to address these issues simultaneously. Cellular reprogramming is an exciting technology that has opened up new avenues for developing a large quantity of personalized stem cells for different therapies [84]. iPS-derived NSCs have been shown to posses tumor homing abilities [85]. Systemic administration of a suicide gene system carrying iPS-derived NSCs has effectively homed to metastatic breast cancer in the preclinical animal model and inhibited tumor growth. However, since iPS can form cancerous teratomas *in vivo*, their application in cell therapies has been limited [86]. One alternative option may be newly developed transdifferentiation technology, where somatic cells can be directly reprogrammed into an alternative lineage by bypassing dedifferentiation step and thus reaching a pluripotent state. To date, using such transdifferentiation method has generated NSCs and several studies confirmed that these NSCs are not tumorigenic [87,88]. Moreover, these NSCs can survive long term *in vivo* and can express high levels of engineered transgene [87,88,89]. Induced stem cells are a viable option as cell carriers for oncolytic virotherapy that require further research to evaluate the feasibility of the reprogramming technology to develop personalized stem cell-based cell carriers for oncolytic virotherapy.

*Reduce premature cell lysis in the carrier cells:* One of the important characteristics to function as cell carriers for oncolytic virotherapy is the support of the therapeutic virus replication. This is challenging, as the loading of a replication competent virus can destroy the cell carrier. Such processes could be counterproductive, as any premature carrier cell lysing can drastically reduce the dose of therapeutic virus at the tumor sites. Even though the kinetics of oncolytic adenovirus replication is much slower in the NSC-based cell carrier as compared to tumor cells, at a higher loading dose the therapeutic virus can significantly reduce the viability of NSC-based cell carriers [34,50]. One possible solution may be the use of viral vectors that do not replicate in the stem cell-based carrier but can adhere to the cell surface and eventually be passed on to tumor cells when the cell carrier arrives at the tumor bed. This strategy has been pioneered by Vile and colleagues, who demonstrated that the therapeutic retrovirus reversibly attached to a murine T cell carrier could effectively target systemic diseases in the murine model [12]. However, this strategy is yet to be evaluated in the stem cell-based carrier. An inducible replication competent viral vector that will allow the turning on of viral replication once carrier cells reach the tumor site may be very useful to overcome this problem. A vitamin D3 regulated oncolytic adenovirus loaded into BM-MSC can significantly improve the targeting efficiency as well as the therapeutic efficacy of MSC-based oncolytic virotherapy [60]. Chiocca and collogues used the FLP-FRT recombinase technology to convert a replication defective vector into a replication competent vector in a regulated fashion [90]. Such approach can be effectively adept to regulate the lytic activity of the OV in the carrier cell and can be turned on only when a sufficient amount of carrier cells reach the distant tumor sites.

*Non-invasive imaging to monitor stem cells in real time:* Finally, real-time dynamic determination of carrier cells’ migratory and distribution kinetics will be critical in optimizing stem cell-based oncolytic virotherapy treatment protocol in the clinical setting. The utilization of non-invasive imaging techniques such as magnetic resonance imaging (MRI) for cellular tracking is a rapidly growing field [34,91]. Protocols have been established where iron oxide-labeled stem cells can be tracked non-invasively in animal models [34,91]. By combining the MRI-based imaging techniques with the histological analysis and the 3D *ex vivo* tissue reconstruction, we have demonstrated that OV loaded NSCs have very similar intratumoral distribution to unloaded NSCs, both displaying about 31% coverage of intracranial tumors in the orthotropic patient-derived glioma xenograft model [92]. Developing such imaging techniques is critical for the tracking of the implanted stem cells in real time, which will allow the evaluation of migratory kinetics as well as portray the intratumoral distribution of therapeutic carrier in real time.

## 7. Concluding Remarks

The conjugal between OV and cell-based carrier concept is an evadible one and has opened up many new avenues for developing a selective approach to target systemic metastasis without affecting normal tissue. Establishing a stem cell-based carrier system with intrinsic tumor homing ability would be the “icing on the cake”, which would allow us to install a GPS system in this anit-cancer biotherapeutic in order to precisely track systemic diseases as well as invasive cancer cells that left their original sites. Establishing a carrier system with immunosuppressive properties will allow us to shield the therapeutic payload in the patient bloodstream. Their capacity to guide the therapeutic cargo toward the tumor burden at the distant site will allow us to effectively target the systemic metastasis. As discussed in this review, the early results from the preclinical setting are optimistic, but certain future advancement will be the key for the successful translation of the stem cell-based oncolytic virotherapy in the clinic. The first issue that needs to be addressed is the limited tumor homing ability of the currently available stem cell-based cell carrier. Our understanding of molecular mechanisms governing the intrinsic tumor homing ability of stem cells is limited and thus must be expanded. Knowledge from such research must be cultivated to develop the next generation stem cell-based cell carrier with enhanced tumor tracking ability. Secondly, the OV-mediated toxicity towards the carrier cells needed be circumvented. This is critical for achieving a therapeutic dose of viral payload at the distant tumor sites that can generate a clinically relevant therapeutic efficacy. An inducible system should be considered to regulate replication-mediated oncolysis of the carrier cells so that an adequate number of therapeutic cargos can reach the distant tumor sites. Finally, carrier cell engraftment efficiency must be improved. The use of reprogramming technology to generate autologous stem cells from the patients’ somatic cells may avoid the use of allogeneic stem cell lines that can be rejected very quickly after implantation. However, the reprogramming technology is in its infancy and such technology needs to be further optimized in order to be ready for the clinic. With these advancements, one might envision in the near future oncologists being able to offer personalized, targeted stem cell-based oncolytic virotherapy to their cancer patients with metastatic diseases.
